# Single-Cell Transcriptomic Atlas of Chicken Ovarian Aging and Cancer Drives Prognostic Model Development

**DOI:** 10.3390/cancers18020243

**Published:** 2026-01-13

**Authors:** Guoqiang Zhu, Susanna Chau Yi Wang, Jiliang He, Jiannan Zhang, Mao Zhang, Yajun Wang

**Affiliations:** 1 Key Laboratory of Bio-Resources and Eco-Environment of Ministry of Education, College of Life Sciences, Sichuan University, Chengdu 610065, China; 2021322040037@stu.scu.edu.cn (G.Z.); hejiliang@stu.scu.edu.cn (J.H.); biozhangjn@scu.edu.cn (J.Z.); 2 Animal Disease Prevention and Food Safety Key Laboratory of Sichuan Province, College of Life Sciences, Sichuan University, Chengdu 610065, China; 3 School of Biological, Earth & Environmental Sciences, University of New South Wales, Sydney 2052, Australia; z5522015@ad.unsw.edu.au; 4 Division of Vascular Surgery, Sichuan Academy of Medical Sciences & Sichuan Provincial People’s Hospital, School of Medicine, University of Electronic Science and Technology of China, Chengdu 610072, China

**Keywords:** ovarian cancer, aging, chicken, scRNA, prognostic model

## Abstract

**Background**: Ovarian cancer remains the deadliest gynecologic malignancy, with its progression closely tied to age-associated remodeling of the tumor immune microenvironment. The laying hen serves as a valuable spontaneous model for human ovarian cancer. Its single-cell analyses may provide valuable insights into the immune-related axis linking ovarian aging to carcinogenesis. **Methods**: This study applied single-cell RNA sequencing to profile ovaries from three laying hen groups, including 35-week-old normal ovaries (A35w), 110-week-old normal ovaries (B110w), and 110-week-old ovarian cancer tissues (C110w). Key analyses had UCell-based scoring of senescence-related pathways and cancer hallmarks, differential expression analysis for overlapping dysregulated genes, LASSO regression-based prognostic model construction, and assessment of chemotherapy sensitivity and immune infiltration. **Results**: A comprehensive cellular landscape of chicken ovaries was established, identifying major immune populations including B cells, CD4+ T cells, CD8+ T cells, macrophages, and plasma cells. Senescence-related pathways and cancer hallmarks showed progressive activation in immune cells from A35w to B110w to C110w. A total of 216 genes commonly dysregulated in aging and carcinogenesis, reveal core links between immune dysfunction and malignant transformation. The 20-gene prognostic model derived from these genes stratified human ovarian cancer patients into high-risk and low-risk groups with significant overall survival differences, exhibited robust predictive performance across TCGA, GSE32063, and GSE140082. The model also predicted the differential chemotherapy sensitivity in high-risk and low-risk patients and correlated with specific immune infiltration patterns in the tumor microenvironment. **Conclusions**: Notably, this is the first single-cell RNA sequencing study of chicken ovarian cancer, and we constructed the 20-gene prognostic model for human ovarian cancer using 216 genes that change significantly in immune cells during both ovarian aging and carcinogenesis. This work provides support to establish the hen as a potential preclinical animal model and a translational tool to guide personalized therapy.

## Simple Summary

Ovarian cancer is the deadliest gynecologic malignancy, with its progression closely linked to age-related changes in the ovarian immune microenvironment. The laying hen, as the spontaneous model for human ovarian cancer research, provides a unique opportunity to reveal the relationship between aging and cancer development. This study used single-cell RNA sequencing to analyze ovaries from young healthy hens, elderly healthy hens, and elderly hens with ovarian cancer, aiming to identify genes commonly dysregulated in ovarian aging and carcinogenesis. The findings led to a prognostic model for human ovarian cancer that predicts patient survival and chemotherapy sensitivity, supporting the hen as a preclinical model and providing a tool to guide personalized therapy.

## 1. Introduction

Ovarian cancer is the deadliest gynecological malignancy, with the tumor immune microenvironment (TME) harboring a diverse array of immune cells that play a critical role in its progression [1,2]. These immune cell populations include CD8+ T cells, CD4+ T cells, tumor-associated macrophages (TAMs), B cells and differentiated plasma cells [3]. Their dynamics are functionally distinct and tightly linked to clinical outcomes. In the context of ovarian cancer treatment, immune cells are pivotal in guiding therapeutic decision-making, far surpassing the role of ovarian somatic cells, which primarily provide the genetic context. For example, CD8+ T cell infiltration correlates with improved platinum sensitivity and prolonged survival [4], and PD-L1 expression in immune cells is a validated biomarker for immunotherapy eligibility [5]. Our study aligns with this paradigm by focusing on immune cell gene expression, as it reflects the real-time interplay between tumor and immune systems.

Recently, single-cell RNA sequencing (scRNA-seq) has revolutionized ovarian cancer immunity research, by differentiating between immune cell heterogeneity and functional states in the TME [6,7]. On the other hand, aging constitutes a pivotal risk factor for ovarian carcinogenesis. The age-associated remodeling of the ovarian immune microenvironment acts as a critical mediator linking cellular senescence and malignant progression [8]. This remodeling profoundly alters the functional states of core immune cell populations within the TME, where scRNA-seq provides unprecedented resolution to dissect the age-associated alterations. Among the age-sensitive immune populations, CD8+ T cells exhibit marked functional decline. Aging promotes an accumulation of exhausted subsets characterized by a high expression of inhibitory receptors including PD1 and TOX alongside reduced cytotoxic capacity [9]. ScRNA-seq studies document these phenotypes, identifying stem-like precursor exhausted cells and effector memory subsets whose depletion correlates with poor treatment response [9,10]. These scRNA-seq findings underscore the need to unravel senescence-mediated immune remodeling for targeted therapies [11]. However, while the association between immune aging and ovarian cancer is well established, a critical unresolved issue persists. The continuous transcriptional dynamics of immune cells during the natural transition from physiological ovarian aging to spontaneous carcinogenesis, particularly at the single-cell resolution, has not been systematically elucidated.

The laying hen represents a uniquely valuable spontaneous model for addressing this unresolved issue. Unlike rodent models that require artificial tumor induction, hens are among the few animal models that naturally undergo both complete physiological ovarian aging and spontaneous ovarian cancer development, while exhibiting remarkable conservation in tumor histopathology, age-dependent tumorigenesis, and core molecular pathways when compared to humans [12,13,14,15]. Its spontaneous tumor development avoids artificial induction biases observed in rodent models, and it allows non-invasive monitoring via transvaginal ultrasound, enhancing its translational utility [15,16,17]. Of marked significance, clinically relevant interventions in hens yield outcomes consistent with human studies. For example, oral contraceptives reduce the prevalence of ovarian cancer in hens and this aligns with epidemiological observations in humans [18,19]. Genistein also inhibits the spontaneous development of ovarian cancer and suppresses tumor growth in hens [20,21]. These findings mirror results from preclinical trials in humans.

Studies investigating immune cell dynamics in chicken ovarian disease have identified patterns consistent with those observed in human ovarian cancer. Flow cytometric analyses and immunohistochemical staining have demonstrated increased numbers of CD4+ T cells, CD8+ T cells, and Bu1a+ B cells in ovarian tumors in comparison with the normal ovarian tissues [22]. Late-stage serous tumors exhibit the highest total immune cell infiltration, which is consistent with observations in human high-grade serous ovarian cancer [22]. Additionally, in hens, secondary lymphoid tissues support immune cell activation and trafficking, which is similar to the function of these immune compartments in humans [23].

Aging also modulates immune cell function in hens. T cells isolated from newly hatched chicks display functional immaturity, which is characterized by impaired proliferation and cytokine secretion [24]. Furthermore, the cytotoxic capacity of peripheral blood CD8+ T cells and their secretion of key cytokines such as IL-2 and IFN-γ, change with increasing age, which reflects the age-associated immune remodeling in this species [25]. The PD-1/PD-L1 pathway also exhibits functional conservation between hens and humans [26]. Blockade of this pathway restores the cytotoxic activity of T cells against tumor cells, highlighting the pathway’s relevance for immunotherapeutic research [26].

With these observations, the single-cell transcriptional heterogeneity of age-sensitive immune populations remains unexplored in laying hens [27,28]. Specifically, the core molecular events that drive immune cells from an aged, dysfunctional state to a tumor-promoting phenotype, including shared dysregulated genes between aging and carcinogenesis, have not been defined. This unresolved issue is particularly critical because the hen’s natural progression uniquely enables the identification of causal links between immune aging and carcinogenesis, as opposed to the correlative associations observed in other models. The present study employs scRNA-seq to profile ovaries from hens in three experimental groups, including 35-week-old and 110-week-old normal hens, 110-week-old hens with ovarian cancer. The analyses were aimed to identify the genes commonly dysregulated in ovarian aging and carcinogenesis in hens, supporting the hen as a potential preclinical animal model and a translational tool to guide personalized therapy.

## 2. Materials and Methods

### 2.1. Animal Ethics and Samples

A total of 9 Lohmann Layer strain hens were obtained from RUNFENG Poultry Cooperative (Sichuan, China) and assigned to 3 groups with 3 biological replicates each: A35w (35-week-old normal ovaries), B110w (110-week-old normal ovaries), and C110w (110-week-old ovarian cancer tissues). All animal experiments adhered to the Guidelines for Experimental Animals issued by the Ministry of Science and Technology of the People’s Republic of China and were approved on by the Animal Ethics Committee of Sichuan University (Permission ID: SCU2203012). The identification of ovarian tumors is based on morphological criteria established in previous studies [13], which can be summarized as a marked shrinkage in ovarian volume and the presence of solid nodular outgrowths.

### 2.2. Single-Cell RNA Sequencing Library Preparation

For scRNA-seq, 3 biological replicates from each group (A35w, B110w and C110w) were selected and immediately snap-frozen in liquid nitrogen to preserve RNA integrity. Frozen samples were transported to SHBIO Biotechnology Co., Ltd. (Shanghai, China) for processing. Tissues were dissociated into single-cell suspensions using standard enzymatic dissociation, and scRNA-seq libraries were constructed with the DNBelab C series platform (BGI Genomics, Shenzhen, China) following the manufacturer’s instructions.

### 2.3. Single-Cell Data Processing and Cell Annotation

Raw sequencing reads were quality-controlled using FastQC to filter out low-quality reads and adapter sequences. Clean reads were aligned to the chicken reference genome using STAR, and gene expression matrices were generated using HTSeq. Then, data processing in Seurat (R) included mitochondrial gene proportion calculation and cell filtering (200–6000 detected genes, mitochondrial proportion < 10%). Normalization used NormalizeData (“LogNormalize”, scale factor 10,000); variable features (2000 total) were identified with FindVariableFeatures (“vst”), followed by scaling and PCA. For doublet removal, the Seurat was objected to SingleCellExperiment for conversion and scDblFinder for doublet identification. Only the singlets were retained for further analyses. Post-filtering, dimensionality reduction was repeated with 30 PCs for PCA and Uniform manifold approximation and projection (UMAP). Cell clustering was performed using FindNeighbors (30 PCs) and FindClusters (resolution 0.8). Finally, cell type annotation was performed using canonical markers to assign identities to each cluster.

### 2.4. Senescence and Cancer Hallmark Activity

Senescence and cancer hallmarks herein were derived from several highly cited studies. Senescence hallmarks [29,30] analyzed included REACTOME Cellular Senescence, REACTOME Oncogene Induced Senescence, REACTOME Senescence Associated Secretory Phenotype, REACTOME Oxidative Stress Induced Senescence, and Genes In Senescent Cells by SAUL in CD8+ T cells. Cancer hallmarks [31,32] analyzed included Evading Growth Suppressors, Genome Instability, Sustained Angiogenesis, Evading Immune Destruction, Resisting Cell Death, Replicative Immortality, and Sustaining Proliferative Signaling. Activity of these senescence and cancer hallmarks in immune cells was assessed using UCell, which calculates gene set enrichment scores based on rank-ordered gene expression. Cohen’s d effect size was computed using the effsize package to quantify activity differences between groups (A35w vs. B110w for aging, B110w vs. C110w for carcinogenesis). Positive values indicated an increased activity in the latter group. Nonparametric statistical significance was evaluated using the coin package, and distributions were visualized using ggplot2.

### 2.5. Overlapped Differential Expressed Genes

Differential gene expression (DGE) between groups was analyzed using Seurat’s FindMarkers function with thresholds including log_2_ fold change (log_2_FC) > 0.25 and adjusted *p*-value < 0.05. Volcano plots for DGE results were generated using EnhancedVolcano. Venn diagrams to identify overlapping dysregulated genes across immune cell types (for aging and carcinogenesis) were created using the ggVennDiagram package.

### 2.6. Prognostic Model Derived from Chicken

Overlapping dysregulated genes above were mapped to human homologs. Transcriptome data and overall survival (OS) information for human ovarian cancer patients were downloaded from The Cancer Genome Atlas (TCGA-OV, n = 378) and Gene Expression Omnibus (GEO) datasets GSE32063 (n = 40) and GSE140082 (n = 379). Genes associated with OS were identified in TCGA-OV via the survival package using Cox proportional hazards regression. Dimensionality was reduced to derive a 20-gene prognostic signature via glmnet using LASSO regression. The risk score was a linear combination of gene expression values weighted by LASSO coefficients, i.e., Risk score = (0.138 × SIRT2) + (0.127 × MGP) + (0.068 × DUSP1) + (0.056 × COLEC12) + (0.037 × ANXA2) + (0.037 × GNS) + (0.035 × IL1B) + (0.030 × BAG3) + (0.023 × GPX3) + (−0.001 × MARCKSL1) + (−0.008 × UCHL1) + (−0.010 × EIF5B) + (−0.018 × QPRT) + (−0.080 × SNX22) + (−0.092 × MANF) + (−0.095 × JCHAIN) + (−0.120 × HSP90AA1) + (−0.122 × PDIA4) + (−0.134 × OTUB1) + (−0.158 × FLOT1) + (−0.200 × HSD17B1) + (−0.243 × IGFBP7). Risk scores were calculated for all TCGA-OV patients using the 20-gene model formula (Risk Score = Σ (Gene expression × corresponding Cox coefficient)), and patients were stratified into high- and low-risk groups using the median risk score as the cutoff. Kaplan–Meier (KM) survival curves and log-rank tests compared OS between groups via survminer. Receiver operating characteristic (ROC) curves and area under the curve (AUC) values were used to assess predictive performance at 1, 3, and 5 years via timeROC.

### 2.7. Chemotherapy Sensitivity and Immune Infiltration

Chemotherapy sensitivity (IC_50_ values) for common ovarian cancer drugs (Cyclophosphamide, Etoposide, Gemcitabine, etc.) was assessed using the CTRP2 database and oncoPredict in R. Correlations between gene expression and IC_50_ values were analyzed using Spearman’s rank correlation via the Hmisc package and visualized as heatmaps using pheatmap. Immune cell infiltration in TCGA-OV samples was estimated using xCELL, a gene expression-based deconvolution tool. Correlations between signature genes and immune cell subsets were visualized as heatmaps using pheatmap.

### 2.8. Selected Signature Gene Expression in Chicken Immune Cells

Two key genes (DUSP1 and HSP90AA1) from the 22-gene prognostic signature were further examined to elucidate cross-species expression patterns. Their expression in chicken ovarian immune cells (across A35w, B110w, C110w groups) was visualized via UMAP using Seurat. Quantitative analysis was performed using FetchData in Seurat, with box plots generated using ggpubr. Statistical comparisons between groups were performed using one-way ANOVA, and significance was labeled using ggsignif.

### 2.9. Statistical Analysis

All statistical tests were two-sided, with ρ < 0.05 defined as statistically significant. For differential gene expression analysis, ρ-values were adjusted using the Benjamini–Hochberg method to control false discovery rate. Cohen’s d effect size was used to quantify the magnitude of differences in senescence/cancer hallmark activity between groups. For survival analysis, the log-rank test was employed to compare overall survival between two groups. Correlations were assessed using Spearman’s rank correlation. One-way ANOVA test was used for multi-group comparisons of gene expression. Data manipulation and aggregation were performed via dplyr, purrr, and tidyr in R.

## 3. Results

### 3.1. Single-Cell Landscape of Chicken Ovaries During Aging and Carcinogenesis

**Figure 1:**
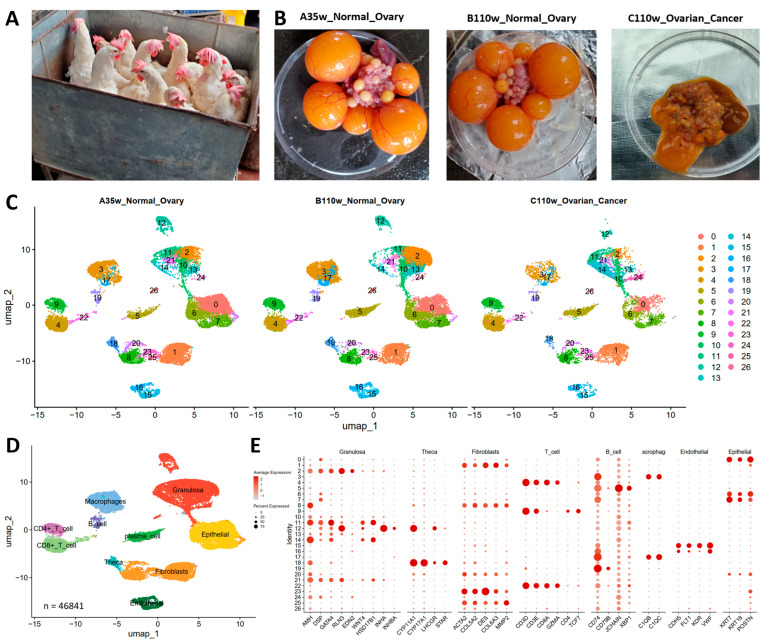
Single-cell landscape of chicken ovaries during aging and carcinogenesis. (**A**) Representative images of experimental chickens from the three experimental groups: 35-week-old normal ovaries (A35w), 110-week-old normal ovaries (B110w), and 110-week-old ovarian cancer tissues (C110w). (**B**) Gross morphology demonstrates typical follicular architecture in A35w and B110w normal ovaries, while C110w tissues exhibit disorganized, tumor-like morphology. (**C**) UMAP visualization of single cells reveals distinct clustering patterns: A35w and B110w show partially overlapping clusters, while C110w displays unique, scattered clusters reflecting tissue heterogeneity. (**D**) Cell type annotation identifies major populations, including granulosa cells (22.3%), epithelial cells (8.7%), fibroblasts (11.4%), macrophages (18.6%), B cells (14.2%), CD4+ T cells (10.1%), CD8+ T cells (9.5%), plasma cells (3.8%), and theca cells (1.4%). (**E**) Dot plot shows canonical marker genes used for cell type annotation.

To investigate immune cell dynamics during ovarian aging and carcinogenesis, we performed scRNA-seq on ovaries from three groups, namely 35-week-old normal ovaries (A35w), 110-week-old normal ovaries (B110w), and 110-week-old ovarian cancer tissues (C110w) (Figure 1A). Gross anatomical observations revealed typical follicular architectures in A35w and B110w normal ovaries, while C110w ovarian cancer tissues exhibited disorganized, tumor-like morphology (Figure 1B). UMAP analysis uncovered distinct cellular clustering patterns across the three groups (Figure 1C). A35w and B110w showed partially overlapping clusters, while C110w displayed unique, scattered clusters that reflected the heterogeneous nature of cancer tissues. Cell type annotation using canonical markers identified major immune cell populations (B cells, CD4+ T cells, CD8+ T cells, macrophages, plasma cells) and ovarian somatic cells (granulosa cells, theca cells, epithelial cells) (Figure 1D,E).

### 3.2. Senescence Pathway Dysregulation in Immune Cells During Carcinogenesis

Cellular senescence-related pathway activity in immune cells was evaluated using UCell score. This analysis first investigated senescence-associated changes between A35w and B110w groups, and this comparison represents the aging process. We then explored whether these pathways also regulate immune cells during carcinogenesis by comparing the RNA expression profiles of B110w and C110w groups. Cohen’s d was used to quantify effect size. Positive values indicate increased pathway activity in the latter group of each comparison.

**Figure 2:**
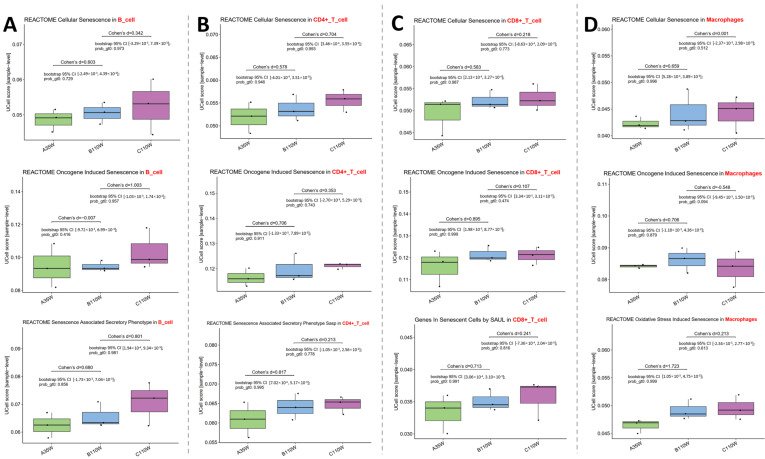
Senescence pathway dysregulation in immune cells during ovarian carcinogenesis. UCell scores reveal progressive activation of senescence-related pathways [29,30] in B cells (**A**), CD4+ T cells (**B**), CD8+ T cells (**C**), and macrophages (**D**) across A35w, B110w, and C110w groups. Identified pathways include REACTOME Cellular Senescence, REACTOME Oncogene Induced Senescence, REACTOME Senescence Associated Secretory Phenotype, Genes In Senescent Cells by SAUL, and REACTOME Oxidative Stress Induced Senescence. Positive Cohen’s d values indicate an increased activity in the latter group.

In B cells (Figure 2A, panel on the far left), REACTOME Cellular Senescence pathway activity increased from A35w to B110w (Cohen’s d = 0.603) and continued to increase from B110w to C110w (Cohen’s d = 0.342). For REACTOME Oncogene Induced Senescence, activity showed minimal difference between A35w and B110w groups (Cohen’s d = −0.007) but was significantly enhanced in C110w when compared to B110w (Cohen’s d = 1.003). The REACTOME Senescence Associated Secretory Phenotype exhibited a heightened activity in B110w when compared to A35w (Cohen’s d = 0.680) and a further increase in C110w vs. B110w (Cohen’s d = 0.801).

In CD4+ T cells (Figure 2B, second panel from the left), REACTOME Cellular Senescence pathway activity was higher in B110w vs. A35w (Cohen’s d = 0.578) and in C110w vs. B110w (Cohen’s d = 0.704). REACTOME Oncogene Induced Senescence activity was elevated in B110w vs. A35w (Cohen’s d = 0.706) and remained higher in C110w vs. B110w (Cohen’s d = 0.353). REACTOME Senescence Associated Secretory Phenotype also showed a stepwise increase, with higher activity in B110w vs. A35w (Cohen’s d = 0.817) and a continued increase in C110w vs. B110w (Cohen’s d = 0.213).

In CD8+ T cells (Figure 2C, third panel from the left), REACTOME Cellular Senescence pathway activity was enhanced in B110w vs. A35w (Cohen’s d = 0.583) and in C110w vs. B110w (Cohen’s d = 0.218). REACTOME Oncogene Induced Senescence activity was higher in B110w vs. A35w (Cohen’s d = 0.895) and showed a modest increase in C110w vs. B110w (Cohen’s d = 0.107). Additionally, the Genes In Senescent Cells by SAUL in the CD8+ T cell pathway exhibited an increased activity in B110w vs. A35w (Cohen’s d = 0.713) and a further increase in C110w vs. B110w (Cohen’s d = 0.241).

In macrophages (Figure 2D, panel on the far right), the REACTOME Cellular Senescence pathway activity increased in B110w vs. A35w (Cohen’s d = 0.659) but showed minimal change in C110w vs. B110w (Cohen’s d = 0.001). REACTOME Oncogene Induced Senescence activity was enhanced in B110w vs. A35w (Cohen’s d = 0.706) but reduced in C110w vs. B110w (Cohen’s d = −0.548). REACTOME Oxidative Stress Induced Senescence also displayed an elevated activity in B110w vs. A35w (Cohen’s d = 1.723) and a continued increase in C110w vs. B110w (Cohen’s d = 0.213), suggesting oxidative stress contributes to immune cell senescence during ovarian carcinogenesis.

### 3.3. Cancer Hallmark Enrichment in Immune Cells During Aging

Cancer-related hallmark activity in immune cells was analyzed via UCell score. This analysis was first used to verify any hallmark activation between B110w and C110w groups, which could provide insights on its association with carcinogenesis. It further explored whether these hallmarks are already pre-enriched between A35w and B110w, and this comparison represents the aging process.

**Figure 3:**
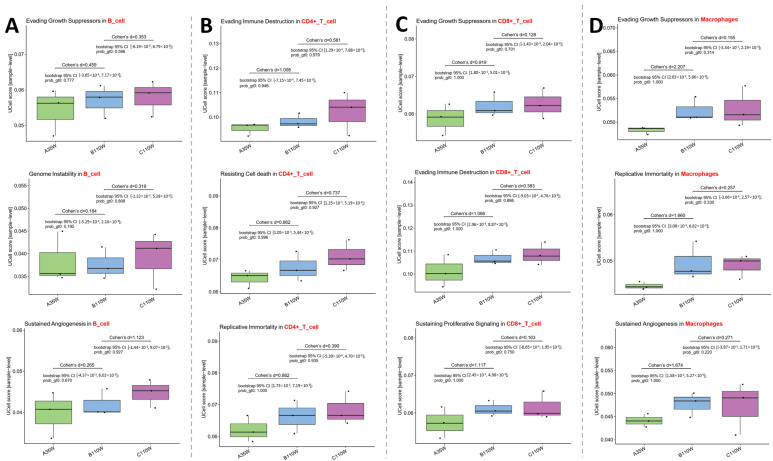
Cancer hallmark enrichment in immune cells during ovarian aging. UCell scores reveal progressive acquisition of cancer hallmarks [31,32] in B cells (**A**), CD4+ T cells (**B**), CD8+ T cells (**C**), and macrophages (**D**) across A35w, B110w, and C110w groups. Identified hallmarks include Evading Growth Suppressors, Genome Instability, Sustained Angiogenesis, Evading Immune Destruction, Resisting Cell Death, Replicative Immortality, Sustaining Proliferative Signaling, and Replicative Immortality. Positive Cohen’s d values indicate increased activity in the latter group.

In B cells (Figure 3A, panel on the far left), Evading Growth Suppressors exhibited increased activity in B110w vs. A35w (Cohen’s d = 0.459) and a further increase in C110w vs. B110w (Cohen’s d = 0.353). Genome Instability showed a modest increase in B110w vs. A35w (Cohen’s d = 0.184) and a more pronounced increase in C110w vs. B110w (Cohen’s d = 0.319). Sustained Angiogenesis displayed elevated activity in B110w vs. A35w (Cohen’s d = 0.265) and a marked increase in C110w vs. B110w (Cohen’s d = 1.123).

In CD4+ T cells (Figure 3B, second panel from the left), Evading Immune Destruction showed a greater increase in B110w vs. A35w (Cohen’s d = 1.008) than in C110w vs. B110w (Cohen’s d = 0.581). Resisting Cell Death exhibited elevated activity in B110w vs. A35w (Cohen’s d = 0.862) and a continued increase in C110w vs. B110w (Cohen’s d = 0.737). Replicative Immortality was enhanced in B110w vs. A35w (Cohen’s d = 0.882) and remained higher in C110w vs. B110w (Cohen’s d = 0.390).

In CD8+ T cells (Figure 3C, third panel from the left), Evading Growth Suppressors showed a notable increase in B110w vs. A35w (Cohen’s d = 0.919) and a slight increase in C110w vs. B110w (Cohen’s d = 0.128). Evading Immune Destruction displayed a strong increase in B110w vs. A35w (Cohen’s d = 1.066) and a modest increase in C110w vs. B110w (Cohen’s d = 0.383). Sustaining Proliferative Signaling exhibited a prominent increase in B110w vs. A35w (Cohen’s d = 1.117) and a slight increase in C110w vs. B110w (Cohen’s d = 0.163).

In macrophages (Figure 3D, panel on the far right), Evading Growth Suppressors showed a striking increase in B110w vs. A35w (Cohen’s d = 2.207) and a modest increase in C110w vs. B110w (Cohen’s d = 0.155). Replicative Immortality displayed a substantial increase in B110w vs. A35w (Cohen’s d = 1.660) and a further slight increase in C110w vs. B110w (Cohen’s d = 0.257). Sustained Angiogenesis exhibited a large increase in B110w vs. A35w (Cohen’s d = 1.674) and a modest increase in C110w vs. B110w (Cohen’s d = 0.271). These data indicate that immune cells progressively acquire cancer hallmarks during ovarian aging and subsequent carcinogenesis.

### 3.4. Overlapping DEGs Between Ovarian Aging and Carcinogenesis

**Figure 4:**
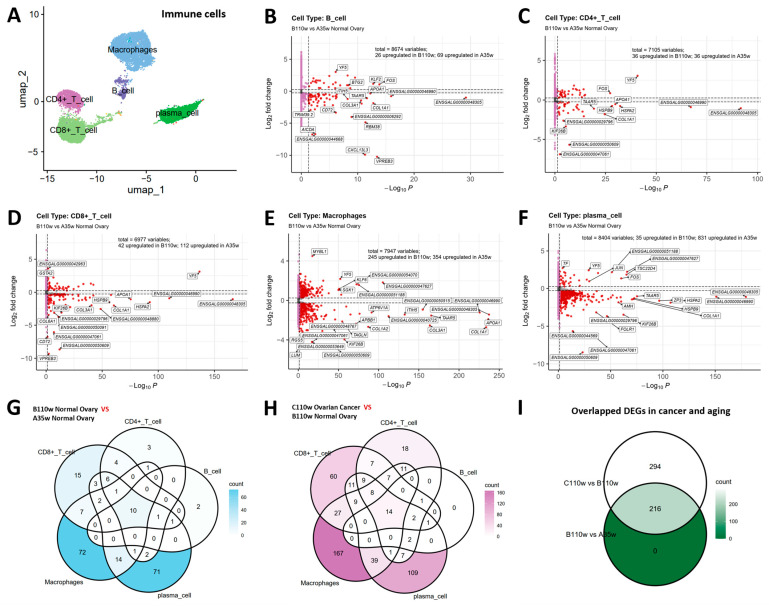
Overlapping DEGs between chicken ovarian aging and carcinogenesis. (**A**) UMAP visualization confirms the distribution of major immune cell subsets (B cells, CD4+ T cells, CD8+ T cells, macrophages, plasma cells). (**B**–**F**) Volcano plots of DEGs in B cells (**B**), CD4+ T cells (**C**), CD8+ T cells (**D**), macrophages (**E**), and plasma cells (**F**) during ovarian aging (B110w vs. A35w). (**G**) Venn diagram reveals 216 genes dysregulated across at least two immune cell types during aging. (**H**) During carcinogenesis (C110w vs. B110w), 510 genes were commonly dysregulated across immune subsets, with 14 shared across all five immune cell types. (**I**) Crucially, 216 genes were commonly dysregulated in both aging and carcinogenesis, representing core molecular links between these processes.

We performed differential gene expression analysis to identify dysregulated genes during ovarian aging (B110w vs. A35w) and carcinogenesis (C110w vs. B110w) in immune cells. UMAP visualization confirmed the distribution of major immune cell subsets (B cells, CD4+ T cells, CD8+ T cells, macrophages, plasma cells) (Figure 4A).

In B cells, 26 genes were upregulated in B110w and 69 genes were upregulated in A35w (Figure 4B). In CD4+ T cells, 36 genes were upregulated in B110w and 36 genes were upregulated in A35w (Figure 4C). In CD8+ T cells, 42 genes were upregulated in B110w and 112 genes were upregulated in A35w (Figure 4D). In macrophages, 245 genes were upregulated in B110w and 354 genes were upregulated in A35w (Figure 4E), while in plasma cells, 35 genes were upregulated in B110w and 831 genes were upregulated in A35w (Figure 4F).

Venn diagrams were used to dissect into the overlapping dysregulated genes across immune cell types for each comparison. For ovarian aging (Figure 4G), the diagram revealed 216 genes dysregulated across at least two immune cell subsets. Notably, 10 genes were commonly dysregulated in all five immune cell types, including ENSGALG00000048305, ENSGALG00000046990, TAAR5, ITIH5, ENSGALG00000040725, ATP6V1A, ENSGALG00000048482, ENSGALG00000051188, KICS2, and ZP3. These pan-immune cell dysregulated genes suggest core molecular events underlying ovarian aging.

For ovarian carcinogenesis (Figure 4H), the Venn diagram showed 510 genes dysregulated across at least two immune cell subsets. Among these, 14 genes were commonly dysregulated in all five immune cell types, including ENSGALG00000027090, ENSGALG00000048305, AMH, ZP3, ENSGALG00000046990, TAAR5, ENSGALG00000040725, ITIH5, ENSGALG00000047727, ATP6V1A, ENSGALG00000048482, ND6, ENSGALG00000051188, and KICS2. The presence of shared dysregulated genes across immune cells highlights conserved molecular alterations during carcinogenesis.

Notably, 216 genes were commonly dysregulated in both ovarian aging and carcinogenesis contexts (Figure 4I), suggesting shared molecular events between ovarian aging and carcinogenesis in immune cells.

### 3.5. Construction and Validation of Human Ovarian Cancer Prognostic Prediction Model

To translate these ovarian findings from chicken to human ovarian cancer mechanisms, we first selected 216 overlapping dysregulated genes from Figure 4I. Among these, 42 genes significantly associated with overall survival were identified using Cox proportional hazards regression. We then performed LASSO regression for dimensionality reduction, yielding a 20-gene prognostic signature.

**Figure 5:**
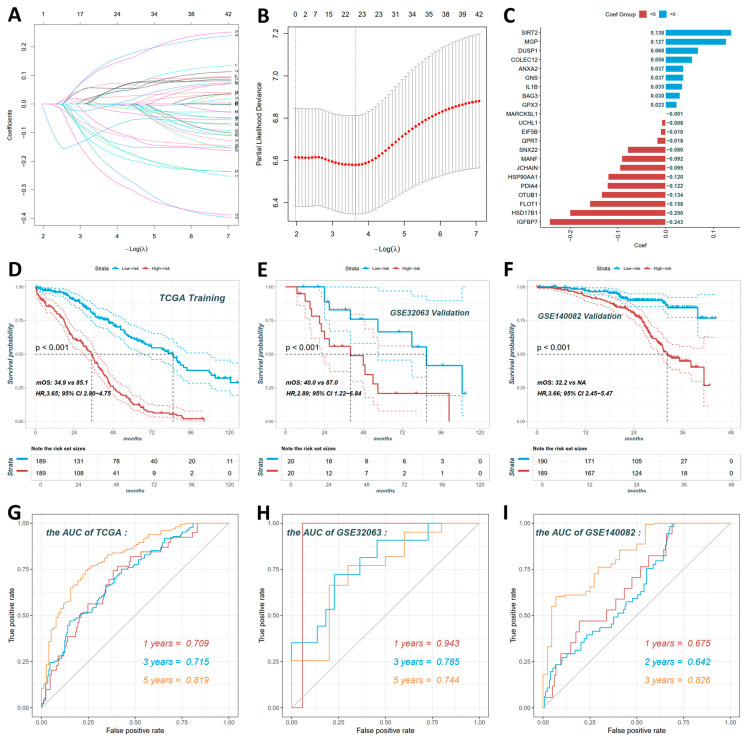
Construction and validation of the 20-gene prognostic model for human ovarian cancer. (**A**) LASSO coefficient profiles for 42 survival-associated genes. (**B**) Cross-validation error curve to select optimal λ for LASSO regression. (**C**) Coefficient distribution of the 20-gene prognostic signature. Risk score formula: Risk score = (0.138 × SIRT2) + (0.127 × MGP) + (0.068 × DUSP1) + (0.056 × COLEC12) + (0.037 × ANXA2) + (0.037 × GNS) + (0.035 × IL1B) + (0.030 × BAG3) + (0.023 × GPX3) + (−0.001 × MARCKSL1) + (−0.008 × UCHL1) + (−0.010 × EIF5B) + (−0.018 × QPRT) + (−0.080 × SNX22) + (−0.092 × MANF) + (−0.095 × JCHAIN) + (−0.120 × HSP90AA1) + (−0.122 × PDIA4) + (−0.134 × OTUB1) + (−0.158 × FLOT1) + (−0.200 × HSD17B1) + (−0.243 × IGFBP7). (**D**) Kaplan–Meier survival curves for high-risk vs. low-risk groups in TCGA training set. High-risk patients (n = 189) had significantly worse OS than low-risk patients (n = 189; HR = 3.65, 95% CI: 2.80–4.75). (**E**) GSE32063 validation set confirmed the prognostic value (HR = 2.89, 95% CI: 1.22–6.84). (**F**) GSE140082 validation set showed consistent results (HR = 3.66, 95% CI: 2.45–5.47). (**G**–**I**) ROC curves for 1-, 3-, and 5-year overall survival prediction in TCGA (**G**), GSE32063 (**H**), and GSE140082 (**I**) datasets.

Figure 5A shows the LASSO coefficient profile, with each curve representing a gene and coefficient magnitude varying with the tuning parameter λ. Figure 5B presents the cross-validation error curve against λ and the optimal λ was used to select the final 20-gene set for the risk model. Figure 5C displays the risk score calculation formula that Risk score = (0.138 × SIRT2) + (0.127 × MGP) + (0.068 × DUSP1) + (0.056 × COLEC12) + (0.037 × ANXA2) + (0.037 × GNS) + (0.035 × IL1B) + (0.030 × BAG3) + (0.023 × GPX3) + (−0.001 × MARCKSL1) + (−0.008 × UCHL1) + (−0.010 × EIF5B) + (−0.018 × QPRT) + (−0.080 × SNX22) + (−0.092 × MANF) + (−0.095 × JCHAIN) + (−0.120 × HSP90AA1) + (−0.122 × PDIA4) + (−0.134 × OTUB1) + (−0.158 × FLOT1) + (−0.200 × HSD17B1) + (−0.243 × IGFBP7).

A risk score for each ovarian cancer patient was computed using a prognostic model. Subsequently, patients were stratified into high-risk and low-risk groups based on their respective scores. In the TCGA training set (Figure 5D), survival analysis revealed high-risk patients had significantly worse overall survival than low-risk patients (log-rank *p* < 0.001; hazard ratio [HR] = 3.65, 95% confidence interval [CI]: 2.80–4.75). The low-risk group had a median overall survival (mOS) of 85.1 months, and the high-risk group had an mOS of 34.9 months. In the GSE32063 validation set (Figure 5E), the prognostic value was confirmed that high-risk patients showed significantly poorer overall survival (log-rank *p* < 0.001; HR = 2.89, 95% CI: 1.22–6.84). The low-risk group had an mOS of 87.0 months, while the high-risk group had an mOS of 40.0 months. In the GSE140082 validation set (Figure 5F), it was consistently observed that high-risk patients had significantly worse overall survival (log-rank *p* < 0.001; HR = 3.66, 95% CI: 2.45–5.47). The low-risk group had an undeterminable mOS, and the high-risk group had an mOS of 32.2 months.

ROC curve analysis further validated the signature’s predictive power, with AUC values demonstrating robust performance across datasets. In TCGA (Figure 5G), AUCs were 0.709 at 1 year, 0.715 at 3 years, and 0.819 at 5 years. In GSE32063 (Figure 5H), AUCs reached 0.943 at 1 year, 0.785 at 3 years, and 0.744 at 5 years. In GSE140082 (Figure 5I), AUCs were 0.675 at 1 year, 0.642 at 2 years, and 0.826 at 3 years.

### 3.6. Chemotherapy Response and Immune Infiltration of the Prognostic Prediction Model

**Figure 6:**
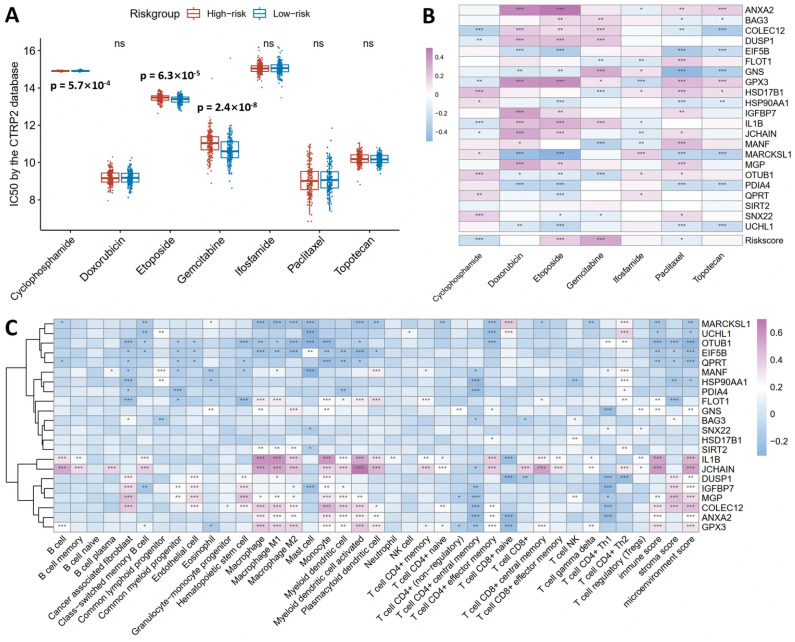
Clinical utility of the 20-gene prognostic model in predicting chemotherapy response and immune infiltration. (**A**) High-risk group showed a significantly lower IC50 for Cyclophosphamide (*p* = 5.7 × 10^−04^), indicating an enhanced sensitivity, but a higher IC50 for Etoposide (*p* = 6.3 × 10^−05^) and Gemcitabine (*p* = 2.4 × 10^−05^), indicating resistance. (**B**) Correlation heatmap shows COLEC12, DUSP1, GPX3, and IL1B positively correlate with Doxorubicin, Etoposide, and Gemcitabine sensitivity, while EIF5B, GNS, HSP90AA1, MARCKSL1, PDIA4, and UCHL1 negatively correlate with Etoposide, Paclitaxel, and Topotecan sensitivity. The risk score negatively correlated with Cyclophosphamide and Paclitaxel IC50 but positively with Etoposide and Gemcitabine IC50. (**C**) Immune infiltration analysis revealed B cells positively correlated with GPX3, IL1B, and JCHAIN; macrophages positively associated with ANXA2, COLEC12, FLOT1, GNS, GPX3, IL1B, JCHAIN, MGP, and SIRT2; CD4+ Th1 cells positively correlated with OTUB1 and SNX22; CD8+ T cells correlated positively with JCHAIN and negatively with BAG3 and DUSP1. Spearman correlation coefficients are color-coded (red: positive, blue: negative). * *p* < 0.05, ** *p* < 0.01, and *** *p* < 0.001, ns means no significance.

To assess chemotherapy sensitivity in human ovarian cancer using the prognostic model, we applied the 20-gene model to TCGA ovarian cancer patients and stratified them into high-risk and low-risk groups. Using the CTRP2 database, we compared IC50 values for common ovarian cancer chemotherapeutics between groups. High-risk patients exhibited a significantly lower IC50 for Cyclophosphamide (ρ = 5.7 × 10^−04^), indicating an enhanced sensitivity to this agent. In contrast, high-risk patients exhibited a significantly higher IC50 for Etoposide (ρ = 6.3 × 10^−05^) and Gemcitabine (ρ = 2.4 × 10^−05^), indicating a reduced sensitivity to both agents (Figure 6A). No significant differences were observed for Doxorubicin, Ifosfamide, Paclitaxel, or Topotecan (Figure 6A).

To shine light on the relationships between individual genes and drug sensitivity, a correlation heatmap (Figure 6B) was constructed. COLEC12, DUSP1, GPX3 and IL1B exhibited positive correlations with sensitivity to Doxorubicin, Etoposide and Gemcitabine. EIF5B, GNS, HSP90AA1, MARCKSL1, PDIA4 and UCHL1 showed negative correlations with sensitivity to Etoposide, Paclitaxel and Topotecan. The risk score itself correlated negatively with Cyclophosphamide and Paclitaxel IC50 and positively with Etoposide and Gemcitabine IC50, validating the model’s capacity to predict differential drug responses.

For immune infiltration (Figure 6C), B cells showed positive correlations with GPX3, IL1B, and JCHAIN and negative correlations with MARCKSL1 and QPRT. And macrophages exhibited positive associations with ANXA2, COLEC12, FLOT1, GNS, GPX3, IL1B, JCHAIN, MGP, and SIRT2 while correlating negatively with EIF5B, MARCKSL1, and OTUB1. As for CD4+ Th1 T cells, they displayed positive correlations with OTUB1 and SNX22 but negative correlations with ANXA2, COLEC12, DUSP1, GNS, IGFBP7, MANF, and MGP. In the case of CD8+ T cells, they correlated positively with JCHAIN and negatively with BAG3 and DUSP1.

### 3.7. Expression Dynamics of Key Genes in Chicken Immune Cells

From the final 20-gene prognostic signature, we selected DUSP1 and HSP90AA1 as representative genes for detailed cross-species validation based on clear expression patterns in chicken ovarian immune cells across A35w, B110w, and C110w groups.

**Figure 7:**
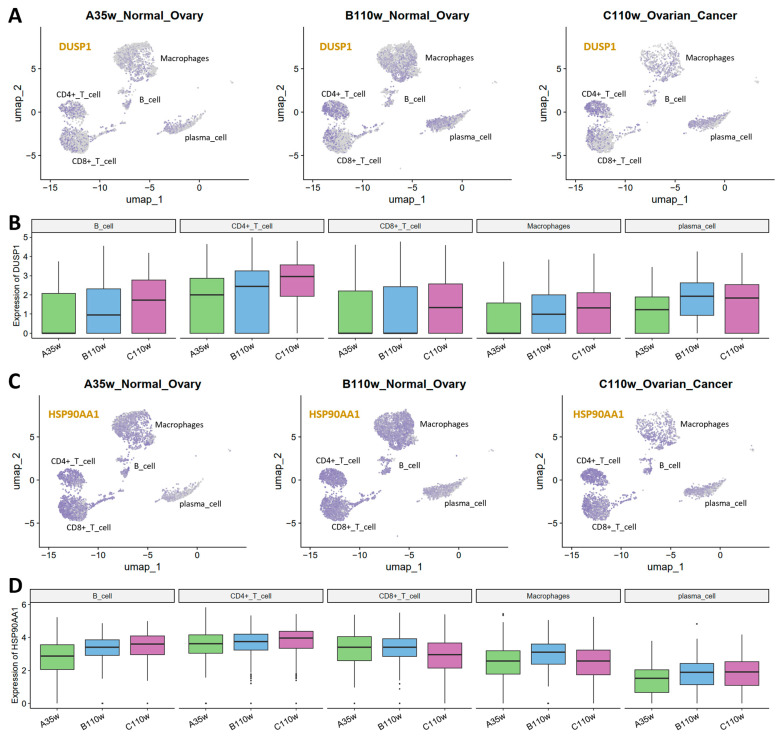
Expression dynamics of key signature genes in chicken immune cells across aging and cancer groups. (**A**) UMAP visualization of DUSP1 expression in A35w, B110w, and C110w immune cells. DUSP1 expression is the most highly enriched in CD4+ T cells across all three groups. (**B**) Box plots of DUSP1 expression across immune cell types (B cells, CD4+ T cells, CD8+ T cells, macrophages, plasma cells) in A35w, B110w, and C110w groups. All immune cells show a gradual increase in DUSP1 expression from A35w to B110w to C110w. (**C**) UMAP visualization of HSP90AA1 expression in A35w, B110w, and C110w immune cells. Similar to DUSP1, HSP90AA1 shows the highest expression in CD4+ T cells across all groups. (**D**) Box plots of HSP90AA1 expression across various immune cell types in A35w, B110w, and C110w groups.

For DUSP1 (Figure 7A,B), UMAP visualization showed the highest enrichment in CD4+ T cells. Quantification revealed DUSP1 expression gradually increased in B cells, CD4+ T cells, and macrophages from A35w to B110w to C110w, while in CD8+ T cells it peaked in C110w. Plasma cells maintained an elevated DUSP1 expression in B110w and C110w when compared to A35w.

For HSP90AA1 (Figure 7C,D), UMAP plots showed the highest expression in CD4+ T cells. B cells, CD4+ T cells, and plasma cells exhibited a slight gradual increase in expression from A35w to B110w to C110w. In contrast, CD8+ T cells showed a slight decrease. Macrophages displayed the highest HSP90AA1 expression in B110w, with low expression and no obvious trend.

## 4. Discussion

To investigate the association between immune cell senescence and ovarian carcinogenesis, this study employed single-cell RNA sequencing to characterize the transcriptomic dynamics of immune cells across three groups of chicken ovaries. These groups comprised 35-week-old normal ovaries (A35w), 110-week-old normal ovaries (B110w), and 110-week-old ovarian cancer tissues (C110w) (Figure 1B). The results established a comprehensive single-cell landscape of chicken ovaries, identifying major immune populations including B cells, CD4+ T cells, CD8+ T cells, macrophages, and plasma cells (Figure 1E). Within these immune cells, we identified 216 genes that are commonly dysregulated during both ovarian aging and carcinogenesis (Figure 4I). This overlapping gene set reflects the core molecular links between immune senescence and malignant transformation. From this set, 22 key genes were further identified using the LASSO regression algorithm to construct a prognostic prediction model for human ovarian cancer (Figure 5C). Built using the TCGA ovarian cancer dataset, the model effectively stratified patients into high- and low-risk groups (Figure 5D). Its prognostic value was validated in the GSE32063 and GSE140082 ovarian cancer datasets, where significant differences in overall survival were observed between the two risk groups (Figure 5E,F). The conserved immune molecular patterns between hens and humans highlight the translational value of the hen model. This suggests that the avian model is a reliable tool for investigating the critical process that transitions from immune abnormalities associated with ovarian aging to carcinogenesis.

Integrated UCell-based scoring of senescence-related pathways (Figure 2) and cancer hallmark enrichment (Figure 3) further reveals progressive molecular changes in immune cells that connect ovarian aging and carcinogenesis. This aligns with previous findings showing that ovarian aging drives gradual immune cell senescence and that this process is exacerbated during carcinogenesis [7,33]. Across B cells, CD4+ T cells, CD8+ T cells, and macrophages, pathways such as cellular senescence and senescence-associated secretory phenotype showed stepwise activation from A35w to B110w and further to C110w (Figure 2). CD8+ T cells exhibited an increased activity of the genes in the senescent cells pathway during aging, and this activity was further enhanced in C110w (Figure 2C). This suggests that age-associated T cell senescence is not static but evolves into more severe dysfunction that supports tumor progression [34]. Macrophages specifically showed robust activation of oxidative stress-induced senescence during aging, and this pattern persisted in C110w (Figure 2D). This aligns with reports that oxidative stress-driven macrophage senescence promotes a tumor-promoting microenvironment due to impaired antigen presentation and heightened pro-inflammatory cytokine secretion [35]. Meanwhile, cancer hallmark enrichment results revealed that ovarian aging itself primes immune cells for malignant transformation. This is supported by studies showing that ovarian aging-induced immune abnormalities create a permissive microenvironment for subsequent tumor initiation [8,36]. Notably, key hallmarks including evading growth suppressors and resisting cell death were already enriched in immune cells during the A35w-to-B110w transition and showed further amplification in C110w (Figure 3). For instance, CD4+ T cells had a marked increase in evading immune destruction activity during aging (Figure 3B), which may weaken anti-tumor surveillance and favor tumor growth [37]. Meanwhile, macrophages showed the most prominent early enrichment of cancer hallmarks including replicative immortality and sustained angiogenesis (Figure 3D). This is consistent with reports that senescent macrophages reshape the ovarian microenvironment to support tumor growth [35,38]. These findings demonstrate that ovarian aging induces progressive molecular and functional reprogramming of immune cells, creating a pre-malignant immune microenvironment that facilitates the transition to carcinogenesis [8,39].

The 216 overlapping differentially expressed genes in immune cells elucidate molecular crosstalk between immune senescence and ovarian malignant transformation. Based on existing literature, these genes can be categorized and associated with aging, cancer, or both. Notably, genes associated with both aging and cancer include BAG3, FLOT1, GPX3, HSP90AA1, IGFBP7, OTUB1, and SIRT2 (Figure 4I and Figure 5C). BAG3 facilitates cancer progression and maintains protein quality control during aging via macro-autophagy pathway recruitment [40,41]. FLOT1 is a novel serum biomarker for ovarian cancer, and its role in immune cell function relates to aging-related immune dysregulation [42]. GPX3 counteracts senescence during adipose tissue remodeling, and its downregulation correlates with poor cancer outcomes in humans [42,43,44]. HSP90AA1 is an unfavorable prognostic factor in hepatocellular carcinoma, and its extracellular form promotes cellular senescence [45,46]. IGFBP7 overexpression ties to poor prognosis and immune infiltration in gastric cancer, and it acts as a key SASP component [47]. It induces senescence in healthy cells via insulin, IGF, and activin A pathway modulation [48]. OTUB1 drives colorectal cancer metastasis and serves as a poor prognostic marker [49]. It inhibits ER-associated degradation of immune checkpoint protein PD-L1 to promote cancer immunosuppression [50]. SIRT2 acts as a tumor suppressor in cancer and its high expression is a novel marker of cellular senescence that depends on wild-type p53 status [51,52]. In addition to these, genes reported to be associated with aging only include DUSP1. DUSP1 promotes intervertebral disc cell senescence via the MYC-DUSP1-P53 axis [53]. Similarly, genes reported to be associated with cancer only can be divided into two categories. The first category includes genes driving cancer progression, such as COLEC12 in gastric cancer [54], HSD17B1 in breast and ovarian cancer [55,56], MGP in colorectal cancer liver metastasis [57], and ZP3 in lung and ovarian cancer [58,59]. The second encompasses genes related to cancer prognosis, including ATP6V1A in gastric cancer [60], and ITIH5 in pancreatic and breast cancer [61,62]. Taken together, these genes drive immune cell reprogramming during ovarian aging to establish a premalignant microenvironment. Their functional associations with aging and cancer strongly suggest key molecular links are shared underlying the two processes, which supports the translational relevance of the hen model for human ovarian cancer research.

The 20-gene prognostic model derived from overlapping immune-dysregulated genes exhibits a robust translational value in human ovarian cancer. Built on the TCGA ovarian cancer training dataset, this model effectively stratifies patients into high-risk and low-risk groups with significantly different overall survival, and its prognostic value is validated in the GSE32063 and GSE140082 datasets (Figure 5). ROC analysis confirms robust predictive performance (Figure 5G–I), aligning with studies validating T cell and macrophage-associated gene profiles as reliable ovarian cancer prognostic tools [63,64,65]. Immune infiltration analysis elucidates the prognostic power of this 20-gene model (Figure 6C). B cells, macrophages, and CD8+ T cells show distinct correlations with genes in the model (Figure 6C), and these patterns mirror reports linking tumor-infiltrating immune cells to ovarian cancer prognosis [66,67]. And the chemotherapy sensitivity analysis extends the clinical utility of this model, with high-risk and low-risk patients exhibiting differential responses to common chemotherapeutics (Figure 6A,B). The translational value of this 20-gene prognostic model is supported by consistent immune related patterns between hens and humans. Comparative transcriptomic studies identified shared pathways between chicken and human ovarian cancer [13]. Progestin lowers ovarian cancer incidence in hens [18], with consistent protective effects reported in humans [19,68]. Curcumin [69], flaxseed [70], and genistein [71] exert anticancer effects in hens, and these findings are mirrored in human studies [72,73,74]. Nanoparticle-based anticancer drugs eliminate tumors in hen models [75], and metformin alters transcriptomic profiles of chicken ovarian cancer cells [76], with human evidence supporting similar potential against ovarian cancer [77]. These results connect immune senescence-driven changes in hens to human ovarian cancer prognosis, supporting the potential of this 20-gene prognostic model for guiding personalized therapy and the role of the hen model in translational ovarian cancer research.

Our study has some limitations. First, our analysis utilized only three animals per experimental group with ovarian cancer samples, which constrains the robustness of complex comparative analyses and broad biological interpretations. Second, our integrated analysis identified evolutionarily conserved gene signatures connecting aging to carcinogenesis, but this exploratory study lacks direct experimental validation of causal mechanisms. Third, while our cross-species prognostic model demonstrates statistically significant associations with survival outcomes in retrospective human datasets, these findings represent preliminary evidence that requires rigorous prospective validation before any clinical application can be considered. For instance, there might be differences in cell composition in the datasets or species-specific gene homology and expression. Based on these limitations, future work employing functional validation and prospective clinical assessment will be essential to confirm our preliminary findings.

## 5. Conclusions

In conclusion, this study provides comprehensive insights into the transcriptomic dynamics of immune cells linking ovarian aging and carcinogenesis through single-cell RNA sequencing in hens. We established a detailed cellular landscape of chicken ovaries, identified 216 conserved dysregulated genes that bridge immune senescence and malignant transformation, and successfully translated these findings into a robust 20-gene prognostic model for human ovarian cancer. This model not only effectively stratifies patient survival risk but also predicts chemotherapy sensitivity and reflects immune microenvironment features, offering a potential tool for guiding personalized therapy. Consistent molecular patterns and therapeutic responses between hens and humans further validate the hen as a reliable preclinical animal model, addressing the gaps in spontaneous ovarian cancer research. Our findings unravel the progressive reprogramming of immune cells during ovarian aging and carcinogenesis, highlight the translational value of cross-species research, and lay a foundation for developing novel preventive and therapeutic strategies for ovarian cancer.

## Data Availability

The single-cell RNA sequencing data from chickens analyzed in this study have been deposited into CNSA (https://db.cngb.org/, accessed on 1 January 2026) with accession number CNP0008144. All data from humans were obtained from The Cancer Genome Atlas (TCGA, https://tcga-data.nci.nih.gov/tcga/, accessed on 1 January 2026) and Gene Expression Omnibus (GEO, https://www.ncbi.nlm.nih.gov/geo/, accessed on 1 January 2026).
